# Initial Health Assessments and HIV Screening under the Affordable Care Act

**DOI:** 10.1371/journal.pone.0139361

**Published:** 2015-09-29

**Authors:** Arleen A. Leibowitz, Agustin T. Garcia-Aguilar, Kevin Farrell

**Affiliations:** 1 Department of Public Policy, UCLA Luskin School of Public Affairs, Box 951656, Los Angeles, CA, 90095–1656, United States of America; 2 AIDS Project Los Angeles, 611 South Kingsley Drive, Los Angeles, CA 90005, United States of America; Public Health Agency of Canada, CANADA

## Abstract

**Background:**

The Centers for Disease Control and Prevention (CDC) estimates that 156,300 (95% CI 144,100–165,900) Americans living with HIV in 2012 were unaware of their infection. To increase knowledge of HIV status, CDC guidelines seek to make HIV screening a routine part of medical care. This paper examines how routinely California primary care providers test for HIV and how providers’ knowledge of California’s streamlined testing requirements, use of sexual histories, and having an electronic medical record prompt for HIV testing, relate to test offers.

**Methods:**

We surveyed all ten California health plans offered under health reform’s Insurance Exchange (response rate = 50%) and 322 primary care providers to those plans (response rate = 19%) to assess use of HIV screening and risk assessments.

**Results:**

Only 31.7% of 60 responding providers reported offering HIV tests to all or most new enrollees and only 8.8% offered an HIV test of blood samples all or most of the time despite the California law requiring that providers offer HIV testing of blood samples in primary care settings. Twenty-eight of the 60 providers (46.6%) were unaware that California had reduced barriers to HIV screening by eliminating the requirement for written informed consent and pre-test counseling. HIV screening of new enrollees all or most of the time was reported by 53.1% of the well-informed providers, but only 7.1% of the less informed providers, a difference of 46 percentage points (95% CI: 21.0%—66.5%). Providers who routinely obtained sexual histories were 29 percentage points (95% CI: 0.2%—54.9%) more likely to screen for HIV all or most of the time than those who did not ask sexual histories.

**Conclusion:**

Changing HIV screening requirements is important, but not sufficient to make HIV testing a routine part of medical care. Provider education to increase knowledge about the changed HIV testing requirements could positively impact testing rates.

## Introduction

The National HIV/AIDS Strategy for the United States set three primary goals: reducing HIV incidence; increasing access to care for people living with HIV (PLWH) and reducing HIV-related health disparities. [1] Providing preventive services for people at high risk for HIV and diagnosing PLWH who are unaware of their HIV infection are key to achieving these goals.

Cost-benefit analyses demonstrate the benefits of routine HIV testing for adults. [2] Early detection and treatment of HIV infection improves the long-term health of people living with HIV (PLWH), and helps to prevent viral transmission to others. Transmission of HIV is reduced by up to 96% when patients’ viral load is suppressed by antiretroviral therapy (ART). [3] Individuals who know their status also reduce high risk behaviors. [4]

The benefits of screening for HIV during routine medical visits were endorsed by the Centers for Disease Control and Prevention (CDC), which released a set of guidelines in 2006 that proposed every patient be screened at least once for HIV and that persons at high risk of HIV infection should be screened at least once a year. [5] The guidelines emphasized incorporating HIV testing as a routine part of medical care and making routine HIV testing more accessible by eliminating requirements known to deter patients from undergoing HIV testing, such as requiring a separate written consent and providing HIV prevention counseling. Instead, general consent for medical care provided in medical settings would be deemed sufficient to encompass consent for HIV screening. [5] The CDC recommended opt-out screenings (rather than asking the patient if they want an HIV test, the provider offers HIV testing, which is carried out unless the patient declines) in all health-care settings.

California passed legislation to implement the CDC recommendations by removing barriers to HIV testing. In 2007, the state passed a law that eliminated the requirement for written consent for an HIV test when ordered by a medical care provider. Since that law went into effect, a verbal consent to HIV testing is sufficient in healthcare settings. [6] Further, pre-test counseling is no longer required. As of January 1, 2014, California also requires that an opt-out HIV test be offered whenever blood is drawn at a primary care visit. [7]

Financial barriers to HIV testing have also been reduced. HIV testing was confirmed as an “essential health benefit” by a 2012 California law. [8] The United States Preventive Services Task Force (USPSTF) places HIV screening of all adults aged 15 to 65 in its “A” group of preventive recommendations, indicating a high degree of certainty that the net benefit in providing this service is substantial. [9] Thus, HIV testing should be free of cost to the patient since the Patient Protection and Affordable Care Act (hereafter ACA) requires insurance plans to cover preventive services rated A or B without any patient cost-sharing. [10]

Despite the advantages of early HIV diagnosis for both individuals and society, the CDC estimated that in 2012 12.8% (95% CI 12.4–13.2%) of those who are living with HIV in the U.S. remained unaware of their infection. [11,12, 13] Although the number of PLWH who are unaware of their infection has fallen from 236,400 (95% CI: 224,900–247,900) in 2008 to 156,300 (95% CI; 144,100–165,900) in 2012 [10, 12] further reductions are vital because this group is estimated to be responsible for about half of the new HIV transmissions. [14]

The implementation of health reform advances the goals of the National HIV/AIDS Strategy by increasing access to routine medical care and reinforcing requirements for preventive care and risk assessments. [1, 15] Health insurance plans offered by Covered California (California’s ACA Health Insurance Exchange) are required to identify and proactively manage all “at-risk” enrollees. [16] All new plans offered under Covered California are managed care plans and all new Medi-Cal (California’s Medicaid program) plans are also organized as managed care and are overseen by the Department of Managed Health Care, which requires that managed care enrollees receive an IHA comprised of “a history and physical examination and an Individual Health Education Behavioral Assessment (IHEBA).”[16] Both HIV screening and initial health assessments (IHAs) are important steps to identify and manage “at-risk” patients because they allow the medical provider to “comprehensively assess the Member’s current acute, chronic, and preventive health needs and identify those members whose health needs require coordination with appropriate community resources and other agencies.” [16]

This paper examines how routinely California primary care providers test for HIV and how testing offers relate to providers’ knowledge of California’s streamlined testing requirements and the requirements of the ACA.

## Methods

In order to understand the challenges of implementing current California law and recommendations regarding HIV screening, we sent surveys to the Medical Director of each of the ten health insurance plans offered under Covered California. The survey asked what information the plan required providers to report on new enrollees and whether the plan recommended obtaining an HIV test on new enrollees. The survey also asked how risk assessment data were used by the plan, whether data were checked for completeness and whether provider groups had reported obstacles in reporting IHAs. The Supplemental materials include copies of the Medical Provider Questionnaire in S1 Text and the_Health Plan Questionnaire in S2 Text.

We also developed a cross-sectional survey on HIV testing and IHAs for primary care providers and piloted it with a convenience sample of Los Angeles medical providers. We developed a one-page survey for primary care providers in four California counties: Alameda, San Diego, Fresno and San Francisco Counties. These counties were selected to represent areas in both northern and southern California with large HIV case loads, as well as a county in Central California with a substantial number of cases, given its size. From the websites of Covered California health plans, we selected primary care providers (family practice and/or internal medicine) serving Covered California enrollees in those counties. Between October 31, 2014 and April 23, 2015, surveys and informed consent materials were faxed to 63 San Diego providers, 61 Alameda providers, 139 Fresno providers, and 59 San Francisco providers.

The one-page survey asked if:

- Questions on sexual health and risk behaviors were included in the IHA
- HIV screening is routinely offered to new enrollees
- Their electronic medical record (EMR) includes a prompt to offer an HIV test
- An HIV test will be done on blood drawn for another purpose, unless the patient objects (Opt-out HIV screening).
- The provider views the following as important barriers to HIV testing: the need for a written informed consent; the need for pre-test counseling; uncertainty regarding reimbursement; competing priorities for time; patient’s anticipated reaction; patients’ sense that they did not need the test; lack of resources for performing the test [17]

We coded providers as not knowledgeable about the California requirements for HIV testing if they incorrectly responded that the need for pre-test counseling or a written consent for an HIV test were important barriers to HIV testing. Other providers were deemed knowledgeable about California HIV testing requirements.

Providers’ responses regarding HIV testing of new enrollees were grouped into three categories: 1) offered all or most of the time; 2) offered sometimes; 3) offered rarely or never. Fisher’s Exact Test was applied to determine whether statistically significant differences in HIV screening and blood testing existed between providers who were more knowledgeable, whose IHA included sexual history, those with an EMR HIV testing prompt; and those lacking these factors. We operationalized “making HIV screening a routine part of medical care” as offering an HIV test to new patients “all or most of the time”.

We calculated differences in the percentage of providers offering an HIV test all or most of the time versus not between providers who had more knowledge of the changes in California requirements and those who had less; between providers whose IHA included questions on sexual history and those whose IHA did not; and between those whose EMR included an HIV test prompt and those who did not. Exact 95% confidence intervals for the difference in routine offer rates were calculated using the riskdiff option in SAS 9.4 PROC FREQ (SAS Institute, Cary NC). [18]

### Ethics Statement

The UCLA Office of the Human Research Protection Program certified the study as exempt on April 25, 2014. (IRB#14–000608).

## Results

### Covered California health plans

Five of the ten health insurance plans available through Covered California responded to our survey. Three companies reported basing their IHA on Covered California guidelines. A fourth plan convened an expert panel to determine IHA content, and a fifth accepted IHAs in whatever format the providers preferred.

Only two of the five plans reported that they required providers to offer each new enrollee an HIV test and to inquire about new enrollees’ HIV status and sexual history. Two of the plans reported that providers rely on EMRs when submitting IHAs. In summary, only Plan #1 appears to conduct comprehensive assessments, supported by EMRs (Table 1).

**Table 1:** Covered California Plans’ Policies Regarding HIV screening of New Enrollees.

Health Insurance Companies	1	2	3	4	5
Does the IHA* require providers to ask new enrollees about their HIV status?	Yes	No	Yes	No	No
Does the IHA require providers to take sexual history for new enrollees?	Yes	No	Yes	No	No
Does the plan ask providers to offer an HIV screening test to each new enrollee?	Yes	No	No	Yes	No
Do you rely on Electronic Medical Records to make your Initial Health Assessments?	Yes	Yes	No	No	No

*IHA = Initial Health Assessment

None of the responding health plans reported imposing penalties on providers for not completing IHAs and only one of the five respondents checked the completeness of risk assessment forms submitted by their medical providers (Plan #3).

#### Covered California providers

We received responses to the one-page survey from 60 of the 322 providers contacted (19%): 16 of the 61 Alameda County providers (26%), 15 of the 63 San Diego County providers (24%), 19 of the 139 Fresno providers (14%) and 10 of the 59 San Francisco providers (17%).

Even though three out of the five health plans that responded to our survey claim that they decide what information to collect from enrollees based on Covered California guidelines, many medical providers reported being unaware of any specific requirements that plans had regarding IHAs.

Only 6.7% of the 60 respondents reported offering routine HIV testing “all the time” to new enrollees, 25% of providers reported offering routine HIV testing “most of the time,” 35% reported offering the test “sometimes,” 21.7% reported rarely offering the test, and 11.7% reported never offering a routine HIV test to new enrollees. Only 5.3% of respondents reported offering HIV testing “all of the time” when blood was drawn, 3.5% reported offering the HIV test “most of the time,” 12.3% offered the test “sometimes,” 21.1% reported rarely offering the test, and 57.9% reported never offering an HIV test when blood was drawn.

Among the 60 responding providers, 46.6% lacked accurate knowledge about HIV testing requirements (Table 2). Offers of HIV testing differed significantly between providers who had accurate knowledge and those who did not (p = .001). A majority of providers (53.1%) who correctly reported that written informed consents and pre-test counseling were not important barriers to HIV testing, offered HIV screening to new enrollees all or most of the time, compared to 7.1% of providers with inaccurate knowledge. The difference of 46 percentage points (95% CI:21.0%—66.5%) differed significantly from zero (p < .001).

**Table 2:** Routine HIV Testing of New Patients by Primary Care Providers, by Knowledge of HIV Regulations, Sexual History and EMR Prompt.

Indicator	Total	Knows HIV Testing Barriers Reduced		IHA Includes Sexual Health and Risk Assessment		HIV Test Prompt in Electronic Medical Record	
		Yes (%)	No (%)	Yes (%)	No (%)	Yes (%)	No%)
**Routine HIV Testing**							
N (%)	60	32	28	41	16	14	43
% of responses	100%	53.3%	46.6%	71.9%	28.1%	24.6%	75.4%
All/Most	31.7%	53.1%	7.1%	41.5%	12.5%	42.9%	25.6%
Sometimes	35%	25.0%	46.4%	34.1%	31.3%	42.9%	32.6%
Rarely/Never	33.3%	21.9%	46.4%	24.4%	56.3%	14.3%	41.9%
Fisher’s Exact Test		P = .001		P = .048		P = .173	
% point difference in All/Most; 95% CI		46.0% (21.0%-66.5%)		29.0% (0.2%-54.9%)		17.3% (-13.5%-46.5%)	
**HIV Test When Blood Drawn**							
N (%)	60	30	27	38	16	14	40
% of responses	100%	52.6%	47.4%	70.4%	29.6%	25.9%	74.1%
All/Most	8.8%	13.3%	3.7%	10.5%	0.0%	0.0%	10.0%
Sometimes	12.3%	13.3%	11.1%	13.2%	12.5%	21.4%	10.0%
Rarely/Never	78.9%	73.3%	85.2%	76.3%	87.5%	78.6%	80.0%
Fisher’s Exact Test		P = .507		P = .557		P = .306	
% point difference in All/Most; 95% CI		9.6% (-16.4%-35.2%)		10.3% (-18.4%-8.0%)		-9.8% (-39.2%-20.8%)	

Most providers (72%) reported that their IHA included questions on sexual health and risk behaviors. Test offering patterns differed significantly between providers whose IHA included these questions and those whose IHA did not. (p = 0.048). Among providers whose IHA included sexual histories and risk assessments, 41.5% offered HIV testing all or most of the time, compared to 12.5% of providers whose IHA lacked these questions, a difference of 29.0 percentage points (95% CI:0.2%—54.9%).

Only 25% of primary care providers reported that their EMR system included a prompt to offer an HIV test. However, 42.9% providers with an EMR prompt offered HIV testing to new enrollees all or most of the time, compared to 25.6% of the providers without an EMR prompt, a difference of 17.3 percentage points that is not statisticaly significant (95% CI:-13.5%—46.5%) (Table 2). There were no significant associations between the offer to test a blood sample for HIV and the knowledge, IHA, or EMR measures (Table 2).

The most frequent reasons providers gave for not offering an HIV test were that “patients do not feel that they need it,” “competing priorities for time,” and “patient’s reaction to the offer of an HIV test.” (Table 3) Only a small share of providers indicated that structural barriers such as lack of infrastructure deterred them for offering HIV screening.

**Table 3:** Physician Assessments of Importance of Barriers to HIV Testing.

Barrier	Average
Patients do not feel that they need it	**3.4**
Competing priorities for time	**2.8**
Patient's reaction to the offer of an HIV test	**2.6**
Need of Pre-test counseling	**2.2**
Uncertainty regarding reimbursement	**2.2**
Need of a signed consent form	**1.8**
Lack of medical resources/infrastructure	**1.3**
5 = more important, 1 = less important	

## Discussion

Despite the fact that the ACA has lowered the financial barriers for HIV screening and that California legislation has reduced many of the implementation barriers to routine HIV testing in medical settings only 31.7% of 60 providers who responded to our survey reported offering HIV tests to all or most new enrollees. Only 8.8% offered an HIV test of blood samples all or most of the time despite the California law requiring that providers offer HIV testing of blood samples in primary care settings.

One reason that the changes to HIV testing requirements have had minimal influence on HIV testing rates is that many primary care providers are unaware of the changes. In our survey, 28 of the 60 providers (46.6%) who responded were unaware that California had reduced barriers to HIV screening by eliminating the requirement for written informed consent and pre-test counseling. However, providers with more accurate knowledge of screening requirements were more likely to offer HIV testing. More than half (53.1%) of the well-informed providers reported screening new enrollees for HIV all or most of the time as compared to 7.1% of the less informed providers, a difference of 46 percentage points (95% CI: 21.0%—66.5%). In addition, providers who routinely obtained sexual histories were 29 percentage points (95% CI: 0.2%—54.9%) more likely to screen for HIV all or most of the time than those who did not ask sexual histories.

People insured for the first time due to the ACA have often lacked access to routine medical care. They also present a prime target group for HIV screening, since many of the demographic groups most likely to have been previously uninsured are also at heightened risk for HIV. Young men were overrepresented among the uninsured prior to the ACA [19] and males accounted for 80% of all new HIV diagnoses in 2012. [12] Young adults had the highest HIV incidence rates in 2012, and also experienced the greatest growth in incidence between 2008 and 2012, while HIV incidence fell among older adults over this period. [12] African Americans were also disproportionately represented among both the uninsured and PLWH. [11] Thus, new enrollees in Covered California present an unparalleled opportunity to increase the share of PLWH who are aware of their status and to provide services to those who are at risk for HIV.

Despite this opportunity, our survey of Covered California providers in four California counties found that fewer than one-third of providers routinely offered HIV screening to new enrollees all or most of the time. This rate is lower than the 40.5% (95% CI 36.3–44.8) of New York State primary care physicians who reported that they “always or frequently” offer HIV testing to patients new to their practice. [20]

National data suggest that 88% of internists were aware of CDC’s call to make HIV testing a routine part of medical care, [21] but our study showed that knowledge about the particular requirements for HIV testing was not widespread. [22,23] This is similar to findings for New York State, where only 61.4% (95% CI 57.4–65.6) of providers had heard of the state’s change in informed consent requirements, even a year after their adoption in 2010. [20]

Providers who responded to our survey who reported that their IHA included sexual health and risk assessments were more likely to offer HIV testing to new patients all or most of the time. Without taking a sexual history, primary care physicians may be unaware that some of their male patients are at increased risk for HIV due to engaging in sex with other men. Questions about sexual behavior are acceptable to most patients [24], yet 47% of respondents in a nationally representative sample of gay and bisexual men reported that they had never discussed their sexual orientation with a doctor and 56% said that no doctor had recommended that they get tested for HIV. [25]

Many physicians feel uncomfortable discussing HIV and Ayra reports that 71% of physicians would prefer that the patient request an HIV test.[23] However, CDC guidelines recommend that providers offer opt-out testing and not wait for patients to ask for an HIV test. One of the contributions of this study is to identify reasons that physicians do not offer HIV screening. The most frequent reason cited by our respondents was that they (the providers) believed that the patients felt that they did not need it. [26] Indeed, among the nationally representative sample of gay and bisexual men who reported that they had never been tested for HIV, 60% cited not thinking they were at risk as their main reason for not testing [25]. Unless providers open a conversation about sexual behavior and unless they offer HIV screening, patients cannot become better informed about their risk and make an informed choice to test for HIV for themselves. However, a recent New York City survey found that over 90% of the adults surveyed were willing to be screened for HIV if their doctor recommended that everyone get tested for HIV, [27]

### Limitations

Our data were collected within the first year of implementation of the Covered California plans. In future years, IHAs may become more standardized and comprehensive, and opt-out HIV testing may become more frequent. It was impossible to establish causality in this cross-sectional survey. Future research could directly test whether provider education and inserting an HIV screening prompt into the EMR stimulate HIV screening. The small sample size of providers with an EMR prompt hindered finding statistically significant results, even when the point estimates showed large differences. The generalizability of our findings may be limited by our collection of data in four California counties with a high burden of HIV, possibly leading to overestimation of testing rates [28]. In addition, response rates among providers were low, with only half of all health plans offered under Covered California responding. Thus, plan responses may not accurately represent the full ten plans offered. Response rates by primary care providers were also low, as is typical of surveys of physicians. Nonetheless, the survey respondents were largely uniform in their failure to offer opt-out HIV screening and routine HIV blood testing on a regular basis, and in their use of sexual histories.

### Conclusions

Our findings indicate that changing HIV screening requirements is important, but not sufficient to make HIV testing a routine part of medical care as recommended by the CDC. Our results also suggest several options to achieve the goal of increasing the rates of HIV screening in primary care settings,

A first option is to increase efforts to educate providers about the changes in HIV screening requirements and about the requirement to offer to screen blood draws for HIV. Even if HIV testing is provided without cost-sharing, as it is under the ACA, testing is less likely if it is not routinely offered by providers. Ample data document that offering opt-out testing greatly increases early HIV diagnosis rates. [29, 30] Educating providers about the changes to HIV testing requirements could substantially increase HIV test offers because we found that sample respondents who knew that written informed consents and pre-test counseling did not present barriers to HIV testing were more likely to report offering HIV tests all or most of the time, [28,30] The small percentage of providers (9%) who offer to test blood samples for HIV suggests that primary care providers are largely unaware of this law, even 9 months following its effective date of January 1, 2014. The ACA’s organization of individual and small group health insurance plans under the rubric of Covered California provides new opportunities to systematically disseminate this information to medical providers in a way not available when health insurance markets were less formally structured.

Structural changes can reinforce educational efforts to stimulate providers to offer HIV testing. Covered California could be more specific and directive about incorporating into the electronic medical record prompts to offer HIV tests. Although not statistically significant, our data indicated a trend toward higher screening rates when the EMR included a prompt to offer an HIV test.

Our findings suggest that providers whose IHA includes taking a sexual history and performing a risk assessment were more likely to offer an HIV test all or most of the time. Covered California could influence HIV screening offers by stressing that taking a sexual history is an important component of the required health assessment.

Another strategy for increasing the offer of HIV testing is to extend the mandate to offer an opt-out HIV test when blood is drawn to urgent care settings, which serve many patients who do not receive routine primary care.

Increasing HIV screening and risk assessment could dramatically increase the percentage of PLWH who are aware of their infection so that they can engage with HIV treatment and prevent further transmission of the virus. In combination with enhanced HIV risk counseling, these efforts have the potential to improve the health of PLWH and decrease HIV incidence rates.

## Supporting Information

S1 Fig Initial Health Assessment Data. (PDF)

S1 Text Medical Provider Questionnaire. (DOCX)

S2 Text Health Plan Questionnaire. (DOCX)
