# Supplementary material for: Initial Health Assessments and HIV Screening under the Affordable Care Act
Source: PLoS One. 2015 Sep 29;10(9):e0139361. doi: 10.1371/journal.pone.0139361 (PMC4587809; doi:10.1371/journal.pone.0139361)
Supplement: S1 Fig — (PDF) [file pone.0139361.s001.pdf]

Initial Health Data

Provider ID	Routine HIV Testing	HIV Test When Blood Draw	Knows HIV Testing Barriers Reduced	Conducts Sexual Health and Risk Assessment	Has Prompt in Electronic Medical Record
1	All/Most of the time	Rarely/Never	Yes	No	No
2	All/Most of the time	.	Yes	Yes	.
3	All/Most of the time	All/Most of the time	Yes	Yes	No
4	All/Most of the time	Rarely/Never	Yes	Yes	No
5	All/Most of the time	Rarely/Never	Yes	Yes	No
6	All/Most of the time	All/Most of the time	Yes	Yes	No
7	Rarely/Never	Rarely/Never	No	No	No
8	Sometimes	Rarely/Never	No	Yes	No
9	All/Most of the time	.	Yes	Yes	No
10	Sometimes	All/Most of the time	No	.	.
11	All/Most of the time	Rarely/Never	Yes	Yes	.
12	All/Most of the time	Sometimes	No	Yes	Yes
13	Rarely/Never	Rarely/Never	Yes	Yes	No
14	All/Most of the time	All/Most of the time	Yes	Yes	No
15	All/Most of the time	Rarely/Never	Yes	Yes	No
16	Sometimes	Sometimes	No	No	Yes
17	All/Most of the time	Rarely/Never	No	Yes	Yes
18	Sometimes	Rarely/Never	No	Yes	Yes
19	Rarely/Never	Rarely/Never	Yes	Yes	No
20	All/Most of the time	Rarely/Never	Yes	Yes	Yes
21	Sometimes	Rarely/Never	No	Yes	No
22	Sometimes	Rarely/Never	Yes	Yes	No
23	All/Most of the time	Rarely/Never	Yes	Yes	No
24	All/Most of the time	Sometimes	Yes	Yes	No
25	Sometimes	Rarely/Never	No	No	No
26	Rarely/Never	Rarely/Never	No	No	No
27	Sometimes	Rarely/Never	Yes	Yes	No
28	Rarely/Never	Rarely/Never	No	No	No
29	Sometimes	Rarely/Never	No	.	No
30	Rarely/Never	Rarely/Never	Yes	Yes	No
31	All/Most of the time	Sometimes	Yes	Yes	Yes
32	Sometimes	Rarely/Never	Yes	Yes	Yes
33	Rarely/Never	Rarely/Never	No	Yes	No
34	Rarely/Never	Rarely/Never	No	No	No
35	Sometimes	Rarely/Never	Yes	Yes	No
36	Sometimes	Rarely/Never	No	No	No
37	Rarely/Never	Rarely/Never	No	No	No

Initial Health Data

Provider ID	Routine HIV Testing	HIV Test When Blood Draw	Knows HIV Testing Barriers Reduced	Conducts Sexual Health and Risk Assessment	Has Prompt in Electronic Medical Record
38	All/Most of the time	All/Most of the time	Yes	Yes	No
39	Sometimes	Sometimes	No	Yes	No
40	Rarely/Never	Rarely/Never	No	No	Yes
41	All/Most of the time	Rarely/Never	Yes	No	Yes
42	Rarely/Never	Rarely/Never	No	No	No
43	Rarely/Never	Rarely/Never	Yes	Yes	Yes
44	Sometimes	Rarely/Never	No	Yes	Yes
45	Sometimes	Rarely/Never	Yes	Yes	No
46	Sometimes	Rarely/Never	Yes	No	No
47	Sometimes	Rarely/Never	No	Yes	No
48	Sometimes	Sometimes	Yes	Yes	No
49	All/Most of the time	Rarely/Never	Yes	Yes	Yes
50	Rarely/Never	Rarely/Never	No	Yes	No
51	Sometimes	Rarely/Never	No	Yes	No
52	Rarely/Never	.	No	.	No
53	Rarely/Never	Rarely/Never	Yes	Yes	No
54	Rarely/Never	Rarely/Never	No	Yes	No
55	Rarely/Never	Rarely/Never	No	No	No
56	Rarely/Never	Rarely/Never	Yes	Yes	No
57	Rarely/Never	Sometimes	Yes	No	No
58	Rarely/Never	Rarely/Never	No	Yes	No
59	Sometimes	Rarely/Never	Yes	Yes	Yes
60	Sometimes	Rarely/Never	No	No	Yes
