# Supplementary material for: Initial Health Assessments and HIV Screening under the Affordable Care Act
Source: PLoS One. 2015 Sep 29;10(9):e0139361. doi: 10.1371/journal.pone.0139361 (PMC4587809; doi:10.1371/journal.pone.0139361)
Supplement: S1 Text — (DOCX) [file pone.0139361.s002.docx]

**
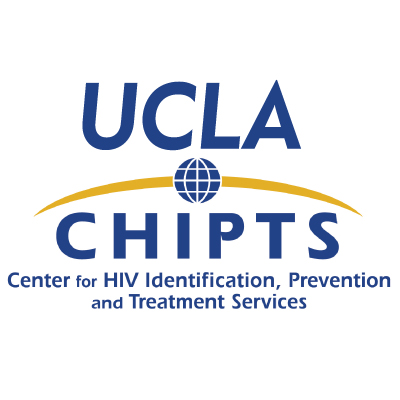
S1 Text Medical Provider Questionnaire**

**SURVEY- MEDICAL PROVIDER GROUPS**

**INITIAL HEALTH ASSESSMENTS**

Survey Code: SSF061

We are interested in learning about the initial health assessments that your medical provider group is required by Covered California health plans to complete for new enrollees to the plan. The focus of this survey is to understand better initial health assessments and potential barriers for HIV screening.

1. Does your Initial Health Assessment include questions on:
   - 1. Physical Health Yes / No
     2. Substance and alcohol use Yes / No
     3. Sexual health and risk behaviors Yes / No
2. Do providers in your medical group routinely offer an HIV screening test to new enrollees?

All the time Most of the times Sometimes Rarely Never

1. Is there a prompt in your electronic medical record to offer an HIV test?

Yes / No

1. When blood is drawn during a visit, will an HIV test be conducted unless the patient objects?

All the time Most of the time Sometimes Rarely Never

1. From 1 (less important) to 5 (more important) how important are the following barriers to offer an HIV test:

Patients do not feel that they need it_____ Need of a signed consent form_____

Need of Pre-test counseling ____ Uncertainty regarding reimbursement_____

Competing priorities for time____ Patient’s reaction to the offer of an HIV test____

Lack of medical resources/infrastructure to offer the test____

***Thank you very much for your time and participation!***
