# Supplementary material for: Initial Health Assessments and HIV Screening under the Affordable Care Act
Source: PLoS One. 2015 Sep 29;10(9):e0139361. doi: 10.1371/journal.pone.0139361 (PMC4587809; doi:10.1371/journal.pone.0139361)
Supplement: S2 Text — (DOCX) [file pone.0139361.s003.docx]

**S2 Text Health Plan Questionnaire**

**SURVEY- HEALTH PLANS**

**INITIAL HEALTH ASSESSMENTS**

We are interested in learning about the initial health assessments that your plan requires of medical providers who are treating new enrollees in your health plan offering through Covered California. The focus of this interview is to obtain information about your standard operating procedures for initial health assessments. In this interview, we ask that you do not reveal the names or identifying information of any patients as doing so could constitute a breach of confidentiality.

1. Please insert the code that was given to you for this survey.
2. Please specify the type of managed health plan that you are working for in your current position:



EPO



HMO



PPO

Other (Please specify):

1. How did your plan decide on what information to collect?



Followed Covered CA guidelines



Convened expert group within the plan to decide



Allowed providers to submit data in the format that was most convenient for them

Other (Please specify):

1. In what format is this information collected and how is it submitted to the plan (e.g., individual level or summary data; electronically?)
2. Does the initial health assessment require providers to take a sexual history for new enrollees?

Yes/No

1. Does the initial health assessment require providers to ask new enrollees about their HIV status?

Yes/No

1. Does the plan ask providers to offer an HIV screening test to each new enrollee?

Yes/No

1. What other kinds of information are required to be collected?
2. Can you give us a copy of the form that you use to collect information for the initial health assessment?

Yes/No

1. How are data from risk assessment questions in the initial health assessment used?
2. Does the plan check for completeness of the forms submitted by medical providers?

Yes/No

1. Does the plan use the data to refer patients to additional supports if they report modifiable risk behaviors (e.g., smoking, overweight)?

Yes/No

1. Are the data used for risk-adjustment across provider groups?

Yes/No

1. What obstacles have provider groups reported to the plan regarding the initial health assessment?
2. What percentage of the provider groups this plan contracts with have reported problems in complying with the initial health assessment?
3. Are these typically large groups or small groups? North or South? Urban or rural?
4. Does the plan impose penalties for not completing initial health assessments?

Yes/No

1. Do you rely on Electronic Medical Records to make your initial health assessments?
2. Can you give us a copy of the list of medical provider groups this plan contracts with?
